# Washington International Congress of Tuberculosis, September-October, 1908

**Published:** 1908-12

**Authors:** 


### Washington International Congress of Tuberculosis, September-October, 1908.

---

**Channels of Entrance of Tuberculosis, by Samuel Bernheim, Paris, President de "l'Oeuvre de la Tuberculose Humaine."**

DOCTOR Bernheim quotes a great number of clinical facts and experiments on animals from which he concludes, contrary to the opinion of Professor Calmette, that tuberculosis is chiefly transmitted by ærial germs and by way of respiratory tract.

1.—The clinical data furnished by pathological anatomy and by experiments since half a century, far from shaking the "ærogenic" doctrine of human tuberculosis, confirm on the contrary this ethiological conception, which sees in the respiratory tract a principal and usual point of entrance of the bacterial contagion.

2.—A great number of cases of tuberculous contagion by the dry bacilli of Koch incorporated in dry dust and numerous other objects, prove the virulence of this microbe even when it is not carried by a moist vehicle. The great facility of realising in man as well as in animals an experimental tuberculosis by inhalation, argues in favor of the view that the respiratory path is a common channel of infection.

3.—The intestine as a door of entrance is possiblė, but it is far from being the normal and usual one. The frequency of human tuberculosis in the countries where the use of milk and meat is very limited, the slight mortality of children by tuberculosis during the period of lactation, demonstrate that their intestines play a role altogether secondary, although appreciable, as a channel of entrance of tuberculosis. Moreover, the necessity of large doses of tuberculous products to produce an experimental tuberculosis and the numerous failures in obtaining it, do not argue in favor of the "enterogenic" doctrine of human tuberculosis.

4.—The bacillary heredity of tuberculosis does not exist, exceptionally it may result from the hereditary contagion "in utero" and cannot be considered as an important factor in the contagion of tuberculosis.

5. The hereditary predisposition must be rejected, for infection in this way is in relation with the degree of "contagiosity" of the environment which produces phthisis, and with the frequency of contaminations.

6.—The vascular path is rare as channel of entrance of Koch's bacillus and it is very difficult to demonstrate its importance.

7.—The genital tract must be considered as a factor of some importance in the propagation of tuberculosis. The clinical observations and experiments demonstrate that this channel of entrance is far from being as rare as has been supposed.

8.—The anal orifice may serve as a channel of entrance to tubercular bacillus in the same conditions as the mucous membranes of the skin. The results concerning that mode of contamination are very numerous, we must then attribute an important ethiological role to this region of human body. The skin may serve as a channel of entrance to tuberculosis in conditions particularly favorable to "contamination" (infective traumatism) nevertheless, it could not be considered as a factor of first importance in the propagation of tuberculosis.

9.—The buccal mucous membrane in the children must be considered as a possible door of entrance for cervical tuberculosis (ganglionary).

---

**Relations of Air with Tuberculosis—Sterilisation of Air.**

From the whole of his work, the author concludes as follows:

1.—There exists a close relation between the microbes of air and tuberculosis. The more is the atmosphere full of bacterias, the greater is the number of tuberculous people living in this unhealthy middle; all conscientious observers have noticed those evident facts. The name of Disease of Darkness has been given to tuberculosis, because microbes in general and particularly the bacilli of Koch, may be kept for a very long period under shelter of light. Under the influence of the normal lifetime of our habits, microbes are cast in the air, men breathe them, his lungs keep them, whence infection and often tubercular contagion.

2.—The absence, or at least the scarcity, of cases of tuberculosis in certain climates (neither altitude or the sea), are again proofs of the close relations between the microbes of the air and tuberculosis. This last disease is an exceptional one in those climates, where a meanest proportion of bacterias do exist. At a certain altitude even no seeds may be found in the air.

3.—For similar or rather inverse reasons a great number of tuberculous people has been observed in the great centers where the air is saturated with bacterias, in overcrowded districts, in places where meetings are held, in hospitals, in prisons, in schools, in barracks, in great shops, in administrations, in mills, in workrooms, and the like.

4.—The best method to get rid of tuberculosis of an aerial origin, is to look after the salubrity of towns, the hygienism of houses where cleanliness must prevail and where solar light must be allowed to penetrate in great abundance. To these natural means certain artificial measures may be added as to lessen the danger caused by aerial bacterias. In fact, by means of certain apparatus of fuel, such as the tub of Dr. Goupil, or Mr. Silbermann's double current chimney, all microbes of the apartments may be destroyed on the spot. In other words, by this method of natural fuel, so full of microörganisms the air may be, it is easy to render it rapidly salubrious.

Those sanitary chimneys may be currently made use of in all apartments, and especially in medicine and surgery rooms, in amphitheatres, in large halls usually crowded and where agglomeration increases the danger of contagion.

---

**Therapeutic Value of Tuberculins.**

Resume of the report delivered at the International Congress of Tuberculosis (Washington, September-October, 1908) by Messrs. Samuel Bernheim, Président "de l'Oeuvre de la Tuberculose Humaine," and P. Barbier, physician to the antituberculosis dispensary for employes of the Post-Office of Paris.

After an historical exposition very much in detail which constitutes a complete review of the scientific researches undertaken during these last years, by the scientists of the whole world with the purpose of discovering a specific remedy for consumption, the authors study the therapeutic value of various methods which have been proposed.

These methods can be divided into two principal groups:

1.—The methods of active immunisation, which necessitates on the part of the organism a reaction of defence ending with the production of specific antibodies.

2.—The methods of passive immunisation not necessitating any reaction on the organism to which are furnished certain ready formed antibodies.

Relying upon the recent works of Bordet on immunity, the authors agree with Professor Sahli in thinking that the future of serumtherapy in a chronic affection like tuberculosis is at the least doubtful.

The methods of active immunisation are themselves divided in two groups: (a) inoculation of bacterial cultures virulent or attenuated—tuberculins properly called: (b) inoculation of soluble products elaborated by microbes—toxins.

Amongst the numerous products proposed for active immunisation, the authors treat only of the most important, those which have been the object of sufficient experimental and clinical researches. They devote to each one a detailed study, examining one after the other, their nature, their mode of preparation, their physiological properties, the doses and the methods of employment, the indications for using and dangers, finally their reciprocal therapeutic value after experimental and clinical data. The authors examine thus in succession:

1.—The first tuberculin of Koch.
2.—The tuberculin T. R. of Koch.
3.—The bacillary emulsion of Koch.
4.—The filtered bouillon of Denys.
5.—The tuberculin of Maréchal.
6.—The tuberculin of Béraneck.
7.—The tuberculin of Jacobs.

Several important facts arise from the clinical study of these various tuberculins. In the first place, this is the necessity of a prudent and methodical dosage of the tuberculin employed. If the Koch's tuberculin has failed this is due to the fact that it has been employed in too strong doses, which have provoked in the patient dangerous reactions which some have believed necessary. All the recent authors (Béraneck, Sahli, Denys, Jacobs) are on the contrary in accord in recognising that one should avoid, as much as possible, every kind of reaction, even the least pronounced, on the patient treated by injections of tuberculins. It is, therefore, necessary to commence with very weak doses, which are raised subsequently in following a gently increasing progression. In this way is opened the antituberculoses immunisation of the organism by a sort of progressive tolerance to the tubercular toxin, by "mithrydatisme" according to the accurate expression of Professor Sahli.

The opsonic method of Wright allows us at present to observe exactly the degree of the immunisation of the organism and the intensity of its defensive reaction in its struggle against bacillus of Koch. This method will, then, be for us a precious guide in deciding the question of the doses to employ and their progressive elevation; thanks to this method, the employment of tuberculin is not an empirical treatment but it is founded upon really scientific basis.

A second fact which results from the comparative study of these different tuberculins, is that all chance of success depends above all upon the state of the organic resistance of the patient. The latter ought to have a sufficient resistance to support the definitive reaction which is going to assure him the final victory in his war against Koch's bacillus. It is again the method of Wright which will inform us upon the value of this organic resistance, and consequently upon the utility of the treatment.

From the comparative study of these different tuberculins, it results finally that it is those which contain the toxins present in the bacilli themselves (tuberculin of Béraneck, and Jacobs), which are capable of possessing the most active immunising properties.

The authors have not had occasion to experiment personally with the Béraneck's tuberculin. As to the tuberculin of Jacobs the very numerous observations (amounting to hundreds) which the authors have collected in their antituberculous dispensaries show at the same time the perfect innocuousness of this product, and its remarkable activity in the most different manifestations of the tubercular infection.

---

In persons with a tendency to hemophilia slight injuries to a joint may be rapidly followed by swelling due to effusion of blood into the synovial sac and severe pain. In such cases there is no rise of temperature nor constitutional disturbances indicating an inflammatory process and the effusion often subsides completely without leaving any traces of its presence.—*International Journal Surgery.*

